# MERTK Inhibition Induces Polyploidy and Promotes Cell Death and Cellular Senescence in Glioblastoma Multiforme

**DOI:** 10.1371/journal.pone.0165107

**Published:** 2016-10-26

**Authors:** Alexandra Sufit, Alisa B. Lee-Sherick, Deborah DeRyckere, Manali Rupji, Bhakti Dwivedi, Marileila Varella-Garcia, Angela M. Pierce, Jeanne Kowalski, Xiaodong Wang, Stephen V. Frye, H. Shelton Earp, Amy K. Keating, Douglas K. Graham

**Affiliations:** 1 University of Colorado Anschutz Medical Campus, 12800 E 19th Avenue, Aurora, CO, 80045, United States of America; 2 Children’s Hospital Colorado, 13123 E. 16th Ave, Aurora, CO, 80045, United States of America; 3 Aflac Cancer and Blood Disorders Center, Children’s Healthcare of Atlanta and Emory University Department of Pediatrics, Atlanta, GA, 30322, United States of America; 4 Winship Cancer Institute, Emory University, Atlanta, GA, 30333, United States of America; 5 Department of Biostatistics and Bioinformatics, Rollins School of Public Health, Emory University, Atlanta, GA, 30333, United States of America; 6 Center for Integrative Chemical Biology and Drug Discovery, Division of Chemical Biology and Medicinal Chemistry, Eshelman School of Pharmacy, University of North Carolina at Chapel Hill, Chapel Hill, NC, 27599, United States of America; 7 Lineberger Comprehensive Cancer Center, Department of Medicine, School of Medicine, University of North Carolina at Chapel Hill, Chapel Hill, NC, 27599, Unites States of America; University of Michigan Medical School, UNITED STATES

## Abstract

**Background:**

MER receptor tyrosine kinase (MERTK) is expressed in a variety of malignancies, including glioblastoma multiforme (GBM). Our previous work demonstrated that inhibition of MERTK using RNA interference induced cell death and chemosensitivity in GBM cells, implicating MERTK as a potential therapeutic target. Here we investigate whether a novel MERTK-selective small molecule tyrosine kinase inhibitor, UNC2025, has similar anti-tumor effects in GBM cell lines.

**Methods:**

Correlations between expression of GAS6, a MERTK ligand, and prognosis were determined using data from the TCGA database. GBM cell lines (A172, SF188, U251) were treated in vitro with increasing doses of UNC2025 (50-400nM). Cell count and viability were determined by trypan blue exclusion. Cell cycle profiles and induction of apoptosis were assessed by flow cytometric analysis after BrdU or Po-Pro-1/propidium iodide staining, respectively. Polyploidy was detected by propidium iodide staining and metaphase spread. Cellular senescence was determined by β-galactosidase staining and senescence-associated secretory cytokine analysis.

**Results:**

Decreased overall survival significantly correlated with high levels of *GAS6* expression in GBM, highlighting the importance of TAM kinase signaling in GBM tumorigenesis and/or therapy resistance and providing strong rationale for targeting these pathways in the clinic. All three GBM cell lines exhibited dose dependent reductions in cell number and colony formation (>90% at 200nM) after treatment with UNC2025. Cell cycle analysis demonstrated accumulation of cells in the G2/M phase and development of polyploidy. After extended exposure, 60–80% of cells underwent apoptosis. The majority of surviving cells (65–95%) were senescent and did not recover after drug removal. Thus, UNC2025 mediates anti-tumor activity in GBM by multiple mechanisms.

**Conclusions:**

The findings described here provide further evidence of oncogenic roles for MERTK in GBM, demonstrate the importance of kinase activity for MERTK tumorigenicity and validate UNC2025, a novel MERTK inhibitor, as a potential therapeutic agent for treatment of GBM.

## Introduction

Glioblastoma multiforme (GBM) is the most common CNS tumor in adults [1]. Patients diagnosed with GBM have a poor prognosis with median survival of ~14 months and a five-year survival rate of less than five percent, even when high dose chemotherapy and radiation are administered. The current standard of care is surgical resection followed by radiation and administration of temozolomide on a cyclic schedule [2]. Genomic analyses of resected GBM patient samples are being used to elucidate subgroups and identify abnormal protein and RNA signatures which could serve as novel therapeutic targets for this dismal disease [3, 4]. Several protein targets with the highest expression or most frequent mutations are currently being validated as therapeutic targets and many newly developed or repurposed targeted agents are being evaluated for in preclinical models. If successful, these agents could be moved forward into clinical trials for patients harboring tumors with specific tumor associated or tumor specific antigens, such as EGFR and EGFR variant III, respectively [5].

There are 58 previously described receptor tyrosine kinases (RTKs), transmembrane proteins that are stimulated by extracellular ligands and activate intracellular pathways. A few of these RTKs have amplified, aberrant, or ectopic expression in GBM tumors, including EGFR, VEGFR, and MERTK [6–8]. MERTK, a member of the TAM family of RTKs, is expressed in ~90% of GBM tumor samples and the majority of GBM cell lines [7]. MERTK is also expressed in other malignancies, including leukemia, melanoma, and non-small cell lung cancer, and mediates activation of proliferative and survival pathways in malignant cells [9–11]. Genetic inhibition of MERTK using small hairpin RNA (shRNA) or small interfering RNA (siRNA) resulted in delayed tumor development, decreased tumor cell proliferation, and induction of apoptosis in GBM and other cancers [7, 12–14]. These data provide rationale for the development of translatable small molecule inhibitors directed against MERTK.

Toward this end, we developed UNC2025, a novel small molecule inhibitor that potently and selectively targets MERTK relative to other TAM family kinases (TYRO 3 and AXL) and does not significantly inhibit PDGF, MET or VEGF receptors [15]. UNC2025 is an ATP competitive class I inhibitor with a *K*_*i*_ for MERTK of ~160 pM. UNC2025 blocks MERTK phosphorylation in cells with an IC_50_ of 2.6 nM, thereby preventing MERTK activation and downstream intracellular signaling. UNC2025 is highly soluble in normal saline and has favorable pharmacokinetic properties in mice [15]. Preclinical evaluation of this compound in non-small cell lung cancer models demonstrated a 50% reduction in tumor cell survival and at 300nM dose abrogates colony formation [16]. Here we evaluated the effects of treatment with UNC2025 in both adult and pediatric GBM cell lines.

## Materials and Methods

### RNA analysis

Level 3 RNAseqV2 data for Glioblastoma Multiforme (GBM) was downloaded from the TCGA data portal. Only patient tumor samples with available clinical and RNAseqV2 expression were analyzed. The gene expression data was log2 transformed and quartile cut-points were applied to compare low (less than first quartile; lower 25%), moderate (greater than equal to first quartile and less than equal to third quartile, middle 50%) and high (greater than third quartile, higher 75%) expression profiles of brain tumor samples for the *GAS6* gene. Log rank test was used to compare overall survival (OS) between the three categories. Pairwise comparisons of OS between categories was done and the adjusted p-values were reported based on the Tukey’s studentized range test. Hazard ratios were calculated using Cox proportional-hazards model and the low expression category as the reference. Pairwise correlation analyses were performed for genes of interest. Survival Analysis and correlation graphs were generated using SAS 9.4 software.

### Cell lines

Adult GBM cell lines U251 and A172 were obtained from American Type Culture Collection (Manassas, VA). Pediatric GBM cell line, SF188, was obtained from UCSF Brain Tumor Bank. Cell lines were maintained in Cellgro^®^ Dulbecco’s Modification of Eagle’s Medium (DMEM) plus 10% fetal bovine serum (standard medium).

### Pharmacologic agents

Lomustine (CCNU), temozolomide and cisplatin were purchased from Sigma-Aldrich^®^ (Sigma Aldrich Corp., St. Louis, MO). Each was reconstituted in dimethyl sulfoxide and stored at -80°C until use. UNC2025, a MerTK tyrosine kinase inhibitor, was synthesized at the University of North Carolina, Chapel Hill as previously described [15]. UNC2025 was reconstituted in dimethyl sulfoxide (DMSO), aliquoted and stored at -80°C until use. UNC2369, a tyrosine kinase inhibitor (TKI) with similar structure to UNC2025 that lacks potent MERTK inhibitory activity (IC50 = 490nM in enzymatic assays versus 0.74nM for UNC2025), was used as a negative control compound.

### Protein lysates and western blotting

Cells were treated with described reagents for the indicated times, then harvested and suspended in lysis buffer (50 mM HEPES, pH 7.5, 150 nM NaCl, 10 mM EDTA, 10% glycerol, 1% Triton X-100, 1 mM Na_3_VO_4_, 0.1 mM Na_2_MoO_4_) with protease inhibitor (Complete Mini, Roche Molecular Biochemicals, Mannheim, Germany) by vortexing. Lysates were incubated on ice for 15 minutes, then centrifuged at 6000rpm for three minutes in a microfuge. Supernatant was collected and stored at -80°C until western blotting. Protein concentrations were determined using the Pierce 660nm Protein Assay (Thermo Fisher Scientific, Rockford, IL). Proteins were resolved by SDS polyacrylamide gel electrophoresis (SDS-PAGE) using the XCell SureLock^™^ Mini-Cell Electrophoresis System with Novex^®^ protein gels. The resolved proteins were transferred to nitrocellulose membranes using the iBlot^®^ dry blotting system (Invitrogen, Carlsbad, CA). Proteins were detected by immunoblot with the following antibodies purchased from Cell Signaling Technology: (phospho-HH3 cat# 9701, Histone H3 cat# 9715, p21 cat# 2947, PARP cat# 9542, phospho-AURKB cat# 3094, AURKB cat# 2914, and α-tubulin cat# 2144). MERTK (cat# ab52968) and TYRO-3 antibodies (cat# ab37841) were purchased from Abcam, and the AXL antibody (cat# AF154) was purchased from R&D.

### Immunoprecipitation

Cells (3 x 10^6^ per sample) were treated with UNC2025 for one hour and 1mM pervanadate was added to the medium for the last five minutes. Cells were harvested and cell lysates were prepared as described above. Lysates were incubated with 3.6ug of MerTK antibody (R&D cat # MAB8912) for 10 minutes and then 25uL of a 50% slurry of recombinant Protein G- Sepharose^®^ beads was added and samples were incubated at 4°C on a rotator overnight. The next day samples were washed and suspended in 2x Laemmli sample buffer, then analyzed by immunoblotting as described above. Membranes were probed with anti-phosphorylated MERTK antibody [16], then bound antibodies were removed from membranes with stripping buffer (H_2_0, 10% SDS, TRIS, β-mercaptoethanol) and membranes were probed with anti-human MERTK antibody (Abcam cat # ab52968). Phosphorylated and total MERTK were quantitated by densitometry using Image J software [17].

### Surface MERTK expression

GBM cells were treated with lomustine, cisplatin or temozolomide for 48 hours. Cells were harvested by incubation in 0.2% EDTA for 20 minutes, then washed with phosphate buffered saline + 2% fetal bovine serum (wash buffer) and blocked for 5 minutes in phosphate buffered saline + 5% fetal bovine serum. Cells were then incubated with anti-human MERTK antibody (hMer 590) [18] for 30 minutes, washed, and then incubated with Allophycocyanin-AffiniPure F(ab')2 Fragment Donkey Anti-Mouse IgG (Jackson Immuno Research, West Grove, PA, cat # 715-136-150) for 30 minutes. Samples were again washed, centrifuged, and resuspended in wash buffer prior to analysis on a Gallios 561 flow cytometer.

### Cell count assay

GBM cell lines were cultured in medium containing 50nM, 100nM, or 200nM UNC2025, or DMSO (vehicle control) for 120 hours, then harvested with 0.25% trypsin and 0.1% EDTA in HBSS (cat # MT-25-053-Cl) purchased from VWR (Radnor, Pennsylvania) and resuspended in 500uL of serum free medium. Cells were then diluted 1:1 in 0.2% trypan blue and counted in duplicate using a Roche Cedex XS Analyzer. To analyze the recovery of cells after drug removal, medium was aspirated from UNC2025 treated cell cultures and fresh medium with or without UNC2025 was applied, and the cultures were incubated for an additional seven days prior to counting as described above.

### Markers of Cellular Senescence

Cells were cultured in a 24-well plate overnight in standard culture medium and then treated with 50nM, 100nM, or 200nM UNC2025 or DMSO for an additional 120 hours. Medium was aspirated and cells were washed, fixed, and stained using a β –Galactosidase Staining Kit (cat # 9860) purchased from Cell Signaling (Danvers, Massachusetts). Cells were observed, photographed, and counted at 400x magnification using a microscope. Approximately one hundred cells were counted per well; when there were fewer than 100 cells per well all cells (minimum = 26) were counted. For assessment of senescence-associated secretory factors, GBM cells were cultured in FBS supplemented medium with UNC2025 or vehicle for 72 hours. Media were collected and stored at -80°C until use. IL-6 and IL-8 was measured using Quantikine human IL-6 and IL-8 ELISAs (R&D Systems, Minneapolis, MN) per manufacturer instructions.

### Cell Cycle analysis

GBM cells were cultured in a 6-well plate overnight. Next day cells were treated with UNC2025 or DMSO for 24, 48, and 72 hours. BD Biosciences (San Jose, California) FITC BrdU Flow kit (cat # 559619) was used for analysis. BrdU (10 uM) was added to culture medium and cells were incubated for an additional 4 hours, then harvested with 0.25% trypsin and 0.1% EDTA in HBSS and fixed and stained according to the protocol provided by the BD Biosciences FITC BrdU Flow kit and analysized on a Gallios 561 flow cytometer. Analysis was performed using Kaluza Software.

### Flow Cytometric Analysis of Po-Pro-1/ Propidium Iodide Incorporation

GBM cells were cultured in medium containing UNC2025 or vehicle for 24, 48 and 72 hours, then harvested with 0.2% EDTA in phosphate buffered saline. Cells were collected by centrifugation and resuspended in PBS containing 2%FBS, 1uM Po-Pro-1 iodide, and 1ug/mL propidium iodide. Fluorescence was measured using a Gallios 561 flow cytometer. Analysis was performed using Kaluza software.

### Clonogenic Assays

Five hundred cells were cultured overnight in standard medium. The next day medium was aspirated and fresh medium containing UNC2025, DMSO, and/or a chemotherapeutic agent was added. After 9 days, the medium was aspirated, and the plates were washed with PBS and then stained with 0.5% crystal violet in 25% ethanol. Colonies were counted using a GelCount colony counter (Oxford Optronix, Oxford, UK).

## Results

### High levels of GAS6 expression are associated with poor prognosis in glioblastoma

**Fig 1:**
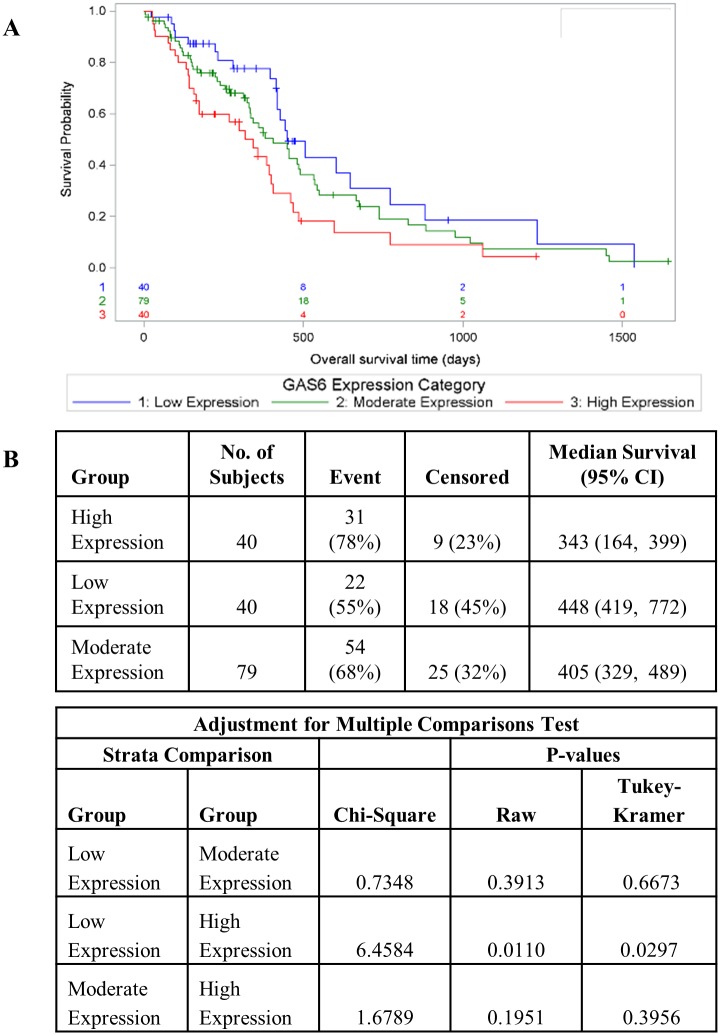
High levels of GAS6 expression correlate with poor prognosis in GBM. GBM samples from the TCGA data base were divided into quartiles based on GAS6 transcript levels and samples with low (bottom quartile), moderate (middle quartiles) and high (top quartile) GAS6 expression were defined. A) Kaplan-Meier curves showing overall survival of GBM patients as a function of GAS6 expression. B) Statistically significant differences were determined using the log rank test and Tukey’s studentized range test was used to adjust for multiple comparisons. Hazard ratios were calculated using Cox proportional-hazards model and the low expression category as the reference.

We and others previously demonstrated co-expression of *MERTK* and *AXL*, *MERTK* and *GAS6* (a TAM kinase ligand [19]), and *AXL* and *GAS6* in tumor biopsies from pediatric patients with glioblastoma multiforme [7, 20]. Here we recapitulated these results using samples from adult patients with GBM from the TCGA database, providing independent confirmation of our previous data and suggesting similar roles for TAM kinase signaling in pediatric and adult GBMs (S1 Fig). The observed co-expression of multiple TAM signaling pathways suggests that dysregulation of non-redundant TAM signaling functions are important for tumorigenesis in GBM. This line of reasoning led us to investigate the relationship between *GAS6* expression, which would be expected to impact signaling through both MERTK and AXL, and tumor progression in GBM using the TCGA database. Patients were divided into quartiles based on *GAS6* transcript levels and assigned to groups with low (lowest quartile), moderate (middle quartiles) and high (top quartile) *GAS6* expression. In these studies, patients with high levels of *GAS6* expression had significantly reduced median survival relative to those with low levels of *GAS6* (343 days post-diagnosis versus 448 days, respectively; p = 0.0110) with a hazard ratio of 2.02 (1.17–3.51 95% CI, p = 0.012) (Fig 1). These data provide evidence of an important role for TAM kinase signaling in GBM tumorigenesis and therapy resistance.

### UNC2025 inhibits MERTK activation and reduces clonal expansion, colony-forming potential, and neurosphere diameter in glioblastoma cells

**Fig 2:**
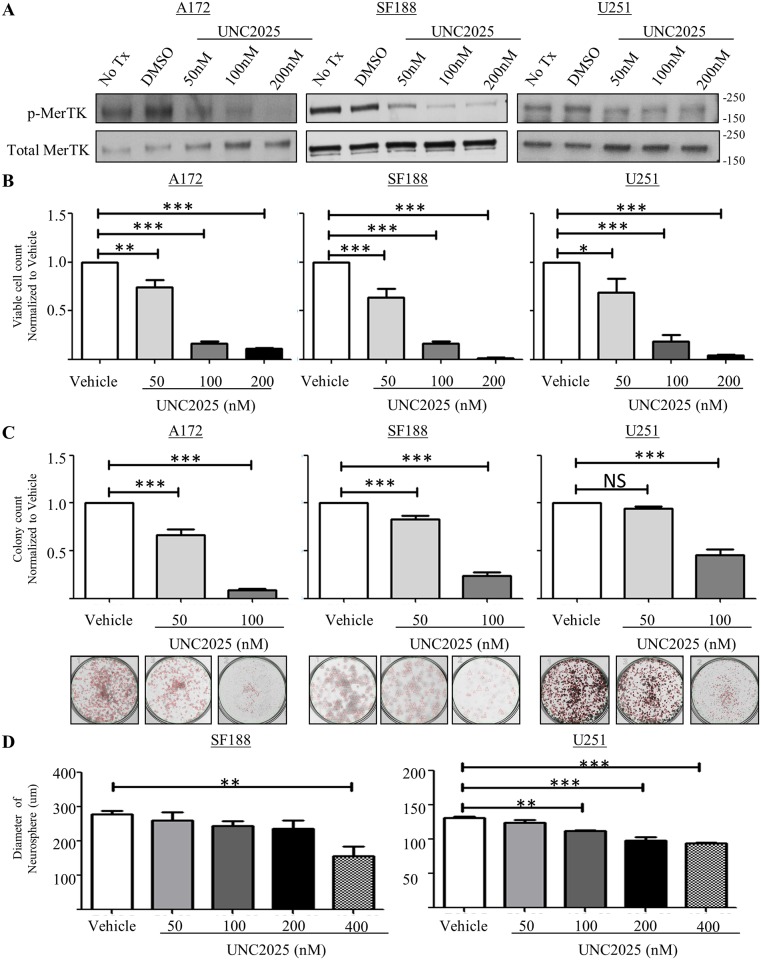
UNC2025 inhibits phosphorylation of MERTK in GBM cells leading to reduced cell number, colony-forming potential, and neurosphere diameter. (A-B) GBM cell lines A172, SF188, and U251, were treated with UNC2025 (50nM, 100nM, 200nM), 200nM UNC2369 (a negative control TKI with similar structure to UNC2025 but lacking MERTK inhibitory activity), or vehicle (DMSO) for one hour. MERTK protein was immunoprecipitated from cell lysates and phosphorylated MERTK protein was detected by immunoblot. (A) Representative images from three independent experiments are shown. (B) Phosphorylated and total MERTK were quantified by densitometry. Mean values and standard errors derived from 3 independent experiments are shown. (C) GBM cell lines were treated with UNC2025 (50nM, 100nM, 200nM) or vehicle (DMSO) for five days and viable cells were counted using trypan blue exclusion. Mean values and standard errors were derived from three independent experiments. (D) GBM cultures were treated with UNC2025 (50nM or 100nM) or vehicle (DMSO) for nine days. Colonies were fixed and stained with 0.5% crystal violet and counted. Mean values and standard errors were derived from six independent experiments. (E) Neurospheres were established for 48 hours and then treated with UNC2025 (50nM, 100nM, 200nM, 400nM) or vehicle (DMSO) for 48 hours. Nine random neurosphere diameters were measured in each well and mean values and standard errors were determined from three independent experiments. (* p<0.5, ** p<0.01, *** p<0.001, 1-sided ANOVA).

We have previously demonstrated that MERTK activates migratory, survival, and proliferative pathways and is critical to GBM tumor growth [7]. the recent development of UNC2025, a highly potent MERTK ATP-competitive type 1 inhibitor, provides a novel and translatable approach to block MERTK activation and test effects on critical malignant phenotypes [15]. To determine the ability of UNC2025 to block MERTK activation in GBM cells, phosphorylation of MERTK was assessed in adult GBM cell lines A172 and U251 and pediatric GBM cell line SF188. After one hour of UNC2025 treatment, MERTK phosphorylation was reduced in all three cell lines and in a dose dependent manner (Fig 2A and 2B).

We have previously reported that reduction of MERTK protein levels following stable transduction with shRNA in the G12 and A172 GBM cell lines reduced colony formation in a soft agar anchorage independent colony forming assay [7]. To determine if UNC2025 mediates a similar phenotypic effect, multiple assays were utilized. First, the relative number of viable cells was determined after five days of treatment with UNC2025 (50nM, 100nM, 200nM) or vehicle (DMSO). In all three GBM cell lines, treatment with UNC2025 significantly reduced the number of viable cells (Fig 2C). The reduction in cell number was dose dependent and the maximum dose of 200nM UNC2025 reduced cell number by 80–98% compared to the vehicle control (DMSO).

To further assess the impact of UNC2025 treatment on GBM cells, equal numbers of sparsely plated GBM cells were treated with UNC2025 (50nM, 100nM, 200nM) or vehicle (DMSO) for nine days and colony number was determined. Treatment with UNC2025 reduced colony formation in a dose dependent manner in all three cell lines (Fig 2D). At the dose of 100nM, UNC2025 colony number was significantly decreased by 60–90% (Fig 2D), with A172 having the largest reduction. Of note, 200nM UNC2025 treatment completely blocked colony formation in all three cell lines. These data demonstrate reduced anchorage-dependent growth and thereby provide evidence of reduced self-renewal of GBM cells mediated by UNC2025.

Relative to two-dimensional cultures, neurosphere formation more closely recapitulates development of a tumor from cancer stem cells [21, 22]. Often the bulk of the tumor is differentiated, though there is variability in molecular expression patterns and some cells are more undifferentiated or “stem-like”. To determine the effect of UNC2025 on stem-like cancer cells, the impact of treatment with UNC2025 on neurosphere formation was determined. Spheres were developed for ~72 hours and treated with UNC2025 (50nM, 100nM, 200nM, 400nM) or vehicle for an additional 48hrs, then the diameter of nine random neurospheres per treatment group was determined and the mean was calculated. Neurospheres derived from the SF188 and U251 cell lines had a significantly reduced diameter in the presence of 400nM UNC2025 (Fig 2E). The A172 cell line did not readily form neurospheres; therefore the assay was not performed with this cell line.

### UNC2025 reduces proliferation and induces polyploidy in glioblastoma cells

**Fig 3:**
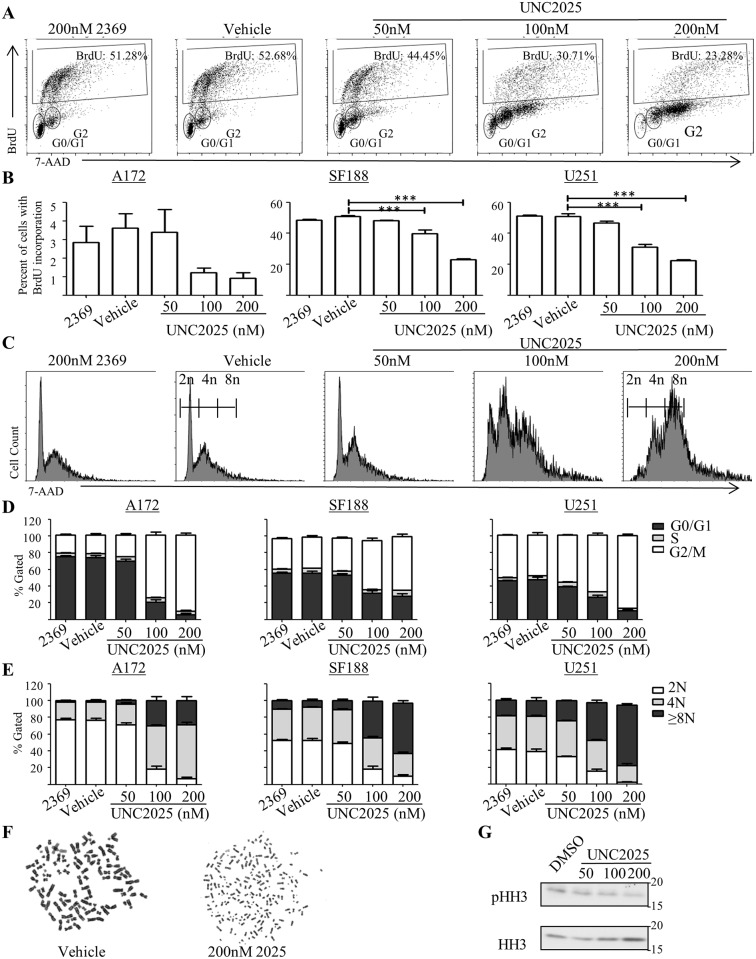
UNC2025 inhibits proliferation and induces polyploidy in GBM cells. A172, SF188, and U251 cells were treated with the indicated concentrations of UNC2025, 200nM UNC2369 (a negative control TKI), or vehicle (DMSO) for 48 hours. (A-C) BrdU was added to the culture medium four hours prior to harvest and cells were fixed, and stained with a fluorescent anti-BrdU antibody and propidium iodide, prior to analysis by flow cytometry. (A) Representative flow cytometry plots showing BrdU incorporation in the U251 cell line. (B) Quantitation of BrdU incorporation. Mean values and standard errors derived from 3 independent experiments are shown. (C) Representative flow cytometry plots showing propidium iodide staining as an indicator of DNA content in the U251 cell line. (D-E) Cells were permeabilized and stained with propidium iodide prior to analysis by flow cytometry. (D) Quantitation of DNA content. Mean values and standard errors derived from four independent experiments are shown. (E) Cell cycle distribution in cells with <8N DNA content. Mean values and standard errors derived from four independent experiments are shown. (F) Metaphase spreads were prepared from U251 cells to determine ploidy. Representative images are shown. (G) Cell lysates were prepared from U251 cells and phosphorylated and total histone H3 (HH3) were detected by immunoblot A representative image from three independent experiments is shown. (* p<0.5, ** p<0.01, *** p<0.001, 1-sided ANOVA).

While it was clear that UNC2025 reduced cell number, it was unclear whether UNC2025 affected proliferation, cell death, or both. The amount of BrdU incorporation significantly decreased following UNC2025 treatment, indicating decreased proliferation in SF188 and U251 cells, while only a trend of decreased proliferation was observed in A172 (Fig 3A and 3B). Additionally, propidium iodide staining, a marker of DNA content, demonstrated a significant population of UNC2025-treated cells with ≥8N DNA content (Fig 3C and 3D, S1A Table). Furthermore, in the population of cells that were not polyploid, there was a significant reduction in G0/G1 phase cells and an increase in G2/M phase (Fig 3E, S1B Table). To confirm increased DNA content in cells treated with the highest dose of UNC2025, metaphase spread was performed on the U251 cell line. As expected, both the untreated and vehicle-treated cells had approximately 4N DNA content (Fig 3F). In contrast, over half of the UNC2025-treated cells had ≥8N DNA content (Fig 3F). To further confirm reduced mitotic activity after exposure to UNC2025, phosphorylated Histone H3 (pHH3), a marker of mitosis, was determined. U251 cells exposed to UNC2025 had reduced levels of pHH3 compared to the vehicle control (Fig 3G).

### UNC2025 induces cell death in glioblastoma cells

**Fig 4:**
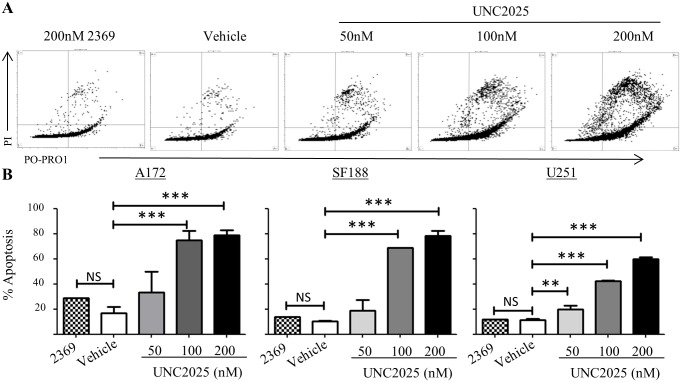
UNC2025 induces cell death in GBM cells. A172, SF188, and U251 cells were treated with UNC2025 (50nM, 100nM, 200nM), control TKI UNC2369 (200nM), or vehicle (DMSO) for 72 hours. Cells were stained with Po-Pro-1 and propidium iodide and analyzed by flow cytometry to determine apoptotic (Po-Pro-1+/PI-) and dead (PI+) cells. (A) Representative U251 flow cytometry profiles. (B) Mean values and standard errors derived from three independent experiments. (* p<0.5, ** p<0.01, *** p<0.001, 1-sided ANOVA).

Prolonged mitotic arrest can induce polyploidy and DNA damage, followed by cell cycle arrest and/or cell death [23]. We have previously shown increased apoptotic cell death, indicated by increased PARP cleavage, in response to shRNA-mediated inhibition of MerTK [7]. To determine whether UNC2025 mediates similar effects, cell membrane disruption was determined as an indicator of cell death. During cellular apoptosis, increased membrane permeability can be detected by assessing infiltration of fluorescent dyes via flow cytometry. Po-Pro-1 is a small dye that can enter the cell during early apoptosis while propidium iodide, a slightly larger dye, is excluded until later stages of cell death. After 72 hours of UNC2025 exposure, A172, SF188, and U251 cells exhibited statistically significant and dose-dependent increases in the fraction of apoptotic and dead cells (Fig 4A and 4B). At the highest dose (200nM) approximately 80% of A172 and SF188 cells and 60% of U251 cells were dead or dying. PARP cleavage, a marker of apoptosis, was also increased in A172 cells and correlated with induction of cell death (S2 Fig). Similarly, treatment with UNC2025 resulted in reduced levels of Survivin, a protein that is downstream of MERTK and functions to inhibit apoptosis (S2 Fig).

### UNC2025 induces cellular senescence in glioblastoma cells

**Fig 5:**
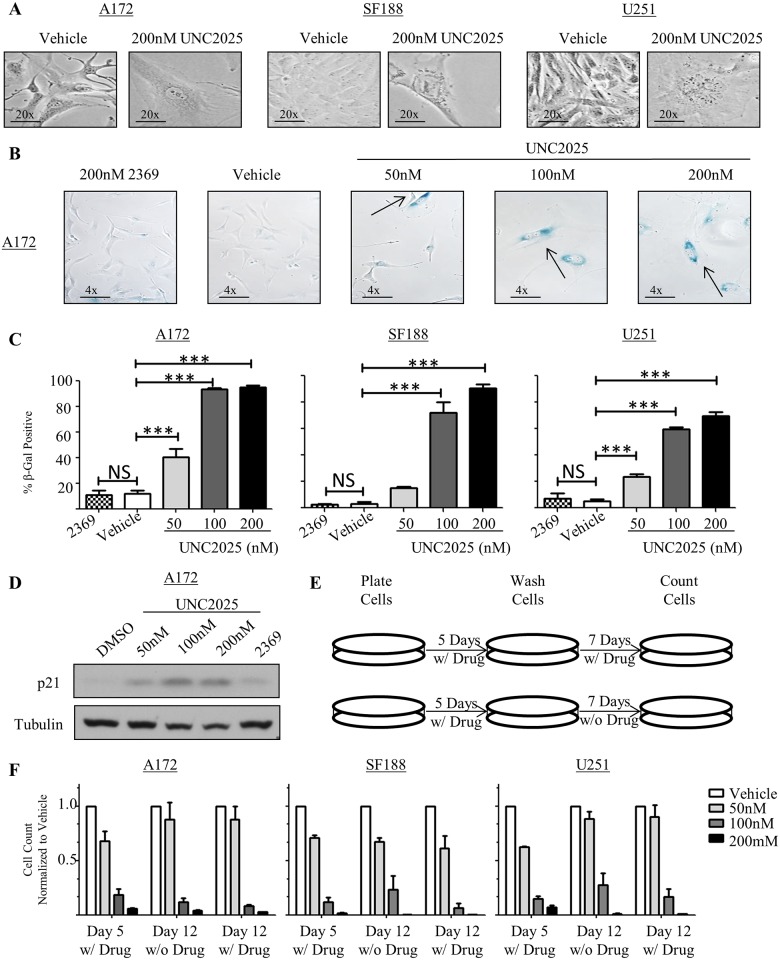
UNC2025 induces cellular senescence in GBM cells. A172, SF188, and U251 cells were treated with the indicated concentrations of UNC2025, UNC2369 control TKI (200nM), or vehicle (DMSO). (A) GBM cells treated with UNC2025 exhibit an altered morphology compared to vehicle (DMSO) treated cells. Representative images from three independent experiments are shown. (B-C) Cells were treated for five days, then fixed and stained to determine beta-galactosidase activity (blue staining = positive). Representative images of the A172 cell line (B) and mean values and standard errors derived from four independent experiments for all three cell lines (C) are shown. (D) A172 cells were treated for 72 hours and p21 protein, a senescence marker, was detected in cell lysates by immunoblot. Representative images from three independent experiments are shown. (E) Schematic of experiment performed in (F). (F) Cells were treated for five days, then media were replaced with fresh medium with or without UNC2025 and cells were cultured for an additional seven days. Viable cells were counted by trypan blue exclusion on days five and twelve. Mean values and standard errors were derived from four independent experiments, statistical analysis revealed no difference between continual drug and drug recovery. (*p<0.5, **p<0.01, ***p<0.001, 1-sided ANOVA).

Visualization of GBM cells after treatment with UNC2025 revealed morphologic abnormalities. Specifically, the surviving adherent cells had a flattened appearance and demonstrated increased cell size and nuclear diameter (Fig 5A). Additionally, the cells appeared to have increased vacuolization. Taken together, these phenotypes are often indicators of cellular senescence [24]. Consistent with this possibility, treatment of A172, SF188, and U251 cells with UNC2025 induced beta-galactosidase activity (blue staining), a prominent marker of senescence.[25] (Fig 5B and 5C). Beta-galactosidase activity was induced in a dose dependent manner, with almost 100% of cells exhibiting beta-galactosidase staining after five days of treatment with the highest dose (200nM) of UNC2025. At a molecular level, p16 and p21 are markers of senescence [24]. GBM cells often have mutations or deletions of cyclin dependent kinase inhibitor 2A (CDKN2A), the gene that encodes p16 and p14. Both the U251 and A172 cell lines have mutated CDKN2A; therefore, p21 expression was investigated as a molecular marker of senescence. Although p21 induction may result from activation of numerous different intracellular pathways, it is best-characterized downstream of p53 so we utilized the A172 cell line, which has wild-type p53, for these studies [24, 26, 27]. Indeed, p21 protein levels were increased in A172 cells upon exposure to UNC2025 (Fig 5D). Similarly, senescence-associated secretory factors IL-6 and IL-8 [28], which are known to be elevated in glioblastoma cells undergoing senescence [29], were significantly elevated by two to three-fold in the A172 and/or U251 cell lines (S3 Fig). A similar trend was observed in SF188 cells.

Cells that have undergone a transient cell cycle arrest may recover and resume proliferation after removal of the initiating stimulus. In contrast, senescence is a stable phenotype and senescent cells do not recover after the initiating treatment is removed [30]. To determine whether the proliferative phenotype observed in GBM cells treated with UNC2025 is reversible, cell cultures were treated with UNC2025 for five days and then the drug was removed and replaced with fresh medium either with or without UNC2025 and expansion of the cells in culture was assessed after an additional seven days (day12 of the experiment) (Fig 5E). Cells exposed to UNC2025 reveal no statistical difference after recovery for seven days in fresh medium without UNC2025 treatment compared to cells continually exposed to UNC2025 (Fig 5F) which is consistent with a senescent phenotype.

Inhibition of Aurora Kinase B (AURKB) phosphorylation and activation has previously been associated with both senescent and polyploid phenotypes [31]. UNC2025 inhibits AURKB in enzymatic assays, although with relatively low potency (IC_50_ = 8.88nM versus 0.46nM for MERTK) [15]. To investigate whether induction of senescence and polyploidy in response to treatment with UNC2025 could be mediated by off-target inhibition of AURKB, GBM cells were treated with UNC2025 and phosphorylation of AURKB was assessed by immunoblot. UNC2025 did not inhibit phosphorylation of AURKB at concentrations sufficient to induce senescence (Fig 5 and S4 Fig).

### MERTK protein levels are increased after cytotoxic therapy

**Fig 6:**
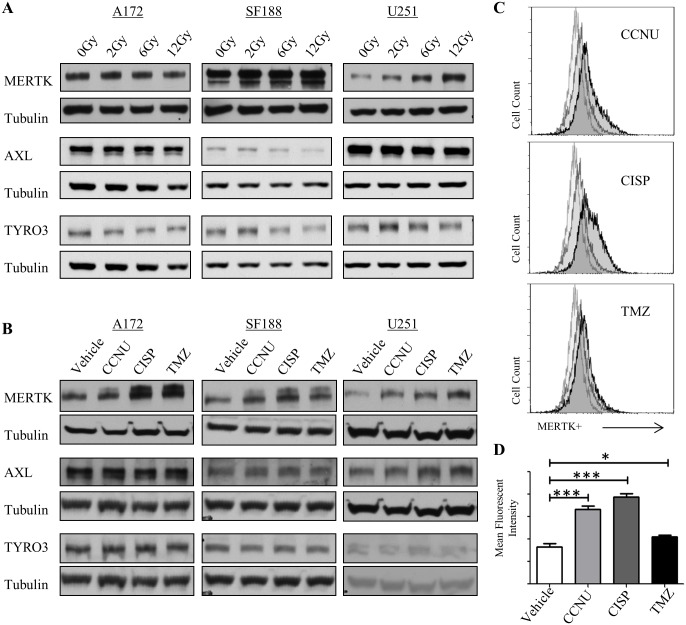
MerTK expression is increased in GBM cells after treatment with radiation and chemotherapy. A172, SF188, and U251 cells were exposed to 50uM lomustine (CCNU), 25uM Cisplatin (CISP), or 250uM Temozolomide (TMZ) (A) or radiation (0Gy, 2Gy, 6Gy, 12Gy) (B) for 48 hours and MERTK, AXL, TYRO3 and tubulin were detected in cell lysates by immunoblot. Representative images from three independent experiments are shown. (C-D) SF188 cells were treated with 50uM lomustine (CCNU), 25uM Cisplatin (CISP), or 250uM Temozolomide (TMZ) as described above and MERTK expression was determined by flow cytometry (colors on the flow cytometry graph; light grey = secondary only; grey = control; black = treatment). Representative images (C) and mean fluorescence intensities (+/- standard error) (D) from 2–4 independent experiments are shown. (* p<0.5, ** p<0.01, *** p<0.001, 1-sided ANOVA).

GBM patients receive temozolomide and radiation (up to 56Gy) as standard therapy.[2] Interestingly, MERTK protein levels were increased in a dose dependent manner in SF188 and U251 cell lines following treatment with cesium source radiation (Fig 6A and S5A Fig). In contrast, MERTK expression was not induced in A172 cells. Similarly, immunoblot analysis revealed a trend toward increased MERTK protein levels in all 3 cell lines following exposure to chemotherapies, including lomustine (CCNU), cisplatin (CISP), and temozolomide (TMZ) (Fig 6B and S5B Fig). Significant increases in MERTK expression were confirmed using flow cytometry to detect MERTK on the surface of SF188 cells treated with cytotoxic chemotherapies (Fig 6C and 6D). AXL and TYRO-3, the other members of the TAM family, did not show any discernable increase after exposure to cytotoxic therapies or radiation (Fig 6A and 6B).

**Fig 7:**
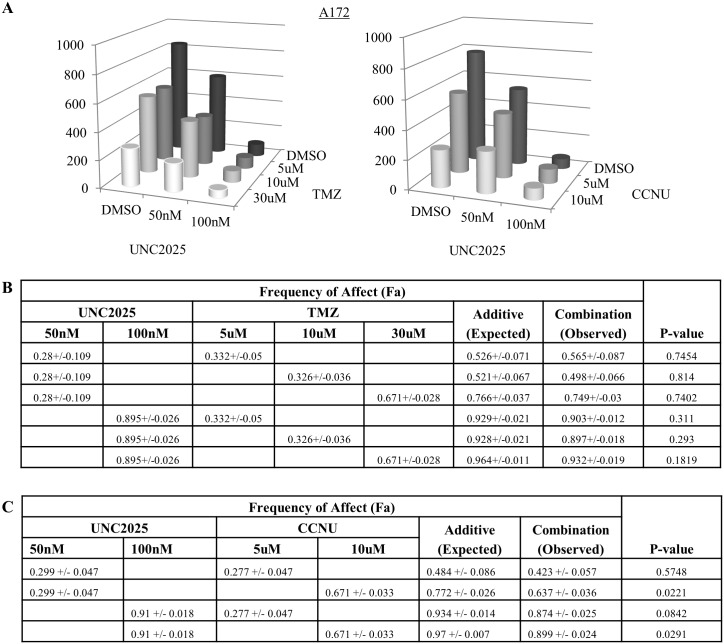
Treatment with UNC2025 in combination with standard GBM chemotherapy is more effective than single agents. A172 cells were incubated for 9 days with UNC2025 or vehicle (DMSO) combined with either lomustine (CCNU) or temozolomide (TMZ). Colonies were fixed and stained with crystal violet in 25% methanol and then counted. (A) Mean values derived from four independent experiments. (B-C) The expected frequency of affect (Fa) for an additive interaction was determined using the Bliss additivity model [32] and is shown (Additive). Statistically significant (p value < 0.05, student’s paired t test) increases in the observed Fa mediated by UNC2025 plus chemotherapy (Combination) relative to the values expected for an additive interaction were not observed, indicating additive interactions. (B) Analysis of interactions between UNC2025 and TMZ. (C) Analysis of interactions between UNC2025 and CCNU.

The up-regulation of MERTK after exposure to traditional GBM therapies suggests that combination therapy might have improved therapeutic effects compared to UNC2025 alone. Consistent with this idea, we previously demonstrated increased chemosensitivity in G12 and A172 GBM cells when transduced with shRNA targeting MERTK [7]. Similarly, GBM cells treated with a combination of UNC2025 and a DNA damaging chemotherapy that is currently used to treat GBM (CCNU or temozolomide) exhibited an additive reduction in colony formation relative to either single agent (Fig 7 and S6 Fig).

## Discussion

Patients with glioblastoma currently suffer a dismal median survival of ~14 months, with only 26% survival at two years [1]. New therapies are needed: novel drugs that decrease tumor cell number by inhibiting proliferation and/or inducing apoptosis. MERTK is expressed in >90% of GBM patient samples, implicating it as a potential target for therapy. Here, we demonstrate decreased survival associated with high levels of *GAS6* expression in GBM, highlighting the importance of TAM kinase signaling in GBM tumorigenesis and/or therapy resistance and providing strong rationale for targeting these pathways in the clinic. Treatment with UNC2025, a novel small molecule inhibitor that potently targets the MERTK ATP binding pocket, reduced phosphorylation (activation) in GBM cell lines. Previous studies demonstrated reduced tumor cell viability and tumor growth in response to inhibition of MERTK using shRNA or a MERTK specific antibody [7, 12, 33]. Here we show similar effects mediated by UNC2025. In GBM cell lines, UNC2025 treatment reduced tumor cell number, colony forming potential, neurosphere diameter, proliferation, and viability, suggesting the utility of UNC2025 as a translational agent and its potential for clinical application.

While UNC2025 induced phenotypes consistent with genetic inhibition of MERTK, upon closer observation we found that exposure to UNC2025 resulted in induction of polyploidy, likely due to endo-reduplication. Mitotic arrest and endo-reduplication creates a highly stressed environment and could be one of the mechanisms leading to induction of cell death in treated cells. Survivin, an anti-apoptotic and pro-proliferative protein, is highly expressed in GBM cells. Previous work from our lab and work shown here demonstrated regulation of survivin protein levels downstream of MERTK in non-small cell lung cancer cells and a reduction in survivin protein in tumor cells treated with UNC2025, including the GBM cell lines U251 and A172 (S5 Fig) [16, 33]. GBMs are known to have chromosomal instability and survivin inhibition in GBM cells results in polyploidy and enlarged cellular size [34]. Survivin forms a complex with aurora kinase B (AURKB) to mediate mitotic spindle assembly checkpoint and cytokinesis functions [35]. Interestingly, while inhibition of Survivin and significant induction of polyploidy were evident at concentrations of UNC2025 as low as 100nM, AURKB phosphorylation was not affected. Taken together, these observations demonstrate induction of polyploidy independent of AURKB inhibition and implicate reduction in survivin in response to treatment with UNC2025 as a mediator of the pleiotropic mitotic defects we observed here, including polyploidy or endo-reduplication and aberrant mitotic spindle formation leading to non-uniform endo-reduplication and eventually to cell death or cellular senescence. In accordance with our findings, previous studies showed that survivin inhibition using RNAi or a pharmacologic agent, YM155, in GBM cell lines was sufficient to induce polyploidy and cell death [36, 37]. Induction of polyploidy in response to MERTK inhibition is a novel finding and reveals a new mechanism by which therapeutic agents targeting MERTK may mediate anti-tumor activity in GBM cells.

Similarly, UNC2025 induces a senescent phenotype in the majority of surviving cells, potentially involving dysregulation of the same AURKB and survivin complex. Previous studies demonstrated induction of a senescent morphology in response to AURKB inhibition [31]. As for the polyploid phenotype, induction of senescence in our studies was independent of AURKB inhibition, suggesting an alternate mechanism for the observed phenotypes. For instance, decreased survivin levels could prevent efficient formation of the survivin/AURKB/INCENP complex, thereby inhibiting nuclear division and cytokinesis independent of any impact on AURKB activity.

We observed higher MERTK protein levels in GBM cell lines in response to treatment with chemotherapy. This observation is consistent with previous data demonstrating increased sensitivity to chemotherapy in GBM cells in response to shRNA-mediated MERTK inhibition and suggests a mechanism by which upregulation of MERTK activates pro-survival signaling pathways to promote chemoresistance. The increase in MERTK could reflect upregulation of the protein in response to chemotherapy treatment to promote tumor cell survival or selective survival of cells expressing higher levels of MERTK protein, providing rationale for MERTK inhibition in combination with cytotoxic chemotherapy for treatment of GBM. Combination therapy is likely to yield the most efficacious results, since single agent therapies have been of little success in patients with GBM thus far. Consistent with this idea, combined treatment with UNC2025 and standard chemotherapies resulted in more efficient inhibition of colony formation relative to single agent therapies.

In summary, MERTK, a transmembrane tyrosine kinase, is overexpressed in GBM cells compared to normal brain tissue, and signals through survival and proliferative pathways. Expression of GAS6, a MERTK ligand, portends poor prognosis, indicating the importance of TAM kinase signaling in patients with GBM. Specific inhibition of MERTK by UNC2025, a small molecule tyrosine kinase inhibitor, reduced GBM cell viability, proliferation, colony-forming potential, and neurosphere diameter and induced polyploidy and senescence. The molecular pathways that are regulated downstream of MERTK inhibition following treatment with UNC2025 to mediate these effects in GBM cells remain to be further elucidated, but the data presented here implicate UNC2025 as a potential therapeutic for treatment of GBM as a monotherapy or in combination with standard therapy.

## Supporting Information

S1 Table Statistical analysis of cell cycle distribution and DNA content in GBM cultures treated with UNC2025, UNC2369, or vehicle. A172, SF188, and U251 were cultured with UNC2025, UNC2369 or DMSO vehicle for 48 hours, then stained with Pro-Po-1 and propidium iodide and analyzed by flow cytometry. (A) Percentages of cells with 2N, 4N, and ≥8N DNA content, determined by 7-AAD staining. (B) Percentages of cycling cells in G0/G1, S, and G2/M phases of the cell cycle were determined based on DNA content indicated by 7-AAD staining. Polyploid cells containing >4N DNA content were excluded from the analysis. Mean values and standard errors were derived from 3 independent experiments. Statistical differences relative to vehicle-treated cultures were determined using 1-sided ANOVA.
(TIF)

S1 Fig Pairwise correlative analysis of AXL, MERTK, and GAS6 transcripts in GBM patient samples. Scatter plots showing significant correlations between expression of MERTK and AXL (left panel), MERTK and GAS6 (middle panel), and AXL and GAS6 (right panel) in GBM patient samples from the TCGA database.
(TIF)

S2 Fig UNC2025 induces PARP cleavage and decreases Survivin expression in GBM cells. A172 cells were cultured with UNC2025 (50nM, 100nM, and 200nM) for 24 (top panels) or 48 (bottom panels) hours. Whole cell lysates were prepared and the indicated proteins were detected by immunoblot. Images are representative of two independent experiments. (FL = Full length).
(TIF)

S3 Fig UNC2025 increases senescence-associated secretory factors IL-6 and IL-8 in glioblastoma cell cultures. The A172, SF188, and U251 cell lines were cultured with 200nM UNC2025 for 5 days, then media was collected and IL-6 and IL-8 proteins were quantitated by ELISA. Mean values and standard errors derived from 3 independent experiments are shown. (*p<0.05, **p<0.01, 1-sided ANOVA)
(TIF)

S4 Fig UNC2025 does not inhibit AURKB at concentrations sufficient to induce senescence in GBM cells. A172 cells were treated with UNC2025 or vehicle for one hour and lysates were prepared. Phosphorylated (denoted by p) and total Aurora Kinase B were detected by immunoblot. Tubulin is shown as a loading control. Images are representative of two independent experiments.
(TIF)

S5 Fig Chemotherapy and radiation increase total MERTK protein levels. Densitometry was used to quantitate immunoblots derived from cells treated with radiation (A) or cytotoxic chemotherapy (B) as depicted in Fig 6. Mean values and standard errors derived from 2–4 independent experiments are shown. (**p<0.01, 1-sided ANOVA)
(TIF)

S6 Fig UNC2025 Exhibits Additive Interactions with Temozolomide and Lomustine in Glioblastoma Cell Lines. SF188 (A-C) and U251 (D-F) were cultured with UNC2025 and/or temozolomide or lomustine (CCNU) for 9 days. Colonies were fixed and stained with crystal violet in methanol, then counted. The expected frequency of affect (Fa) for an additive interaction was determined using the Bliss additivity model [32] and is shown (Additive). Statistically significant (p value < 0.05, student’s paired t test) increases in the observed Fa mediated by UNC2025 plus chemotherapy (Combination) relative to the values expected for an additive interaction were not observed, indicating additive interactions. Mean values and standard errors were derived from 4–6 independent experiments.
(TIF)
