# Supplementary figures and images for: MERTK Inhibition Induces Polyploidy and Promotes Cell Death and Cellular Senescence in Glioblastoma Multiforme
Source: PLoS One. 2016 Oct 26;11(10):e0165107. doi: 10.1371/journal.pone.0165107 (PMC5081168; doi:10.1371/journal.pone.0165107)

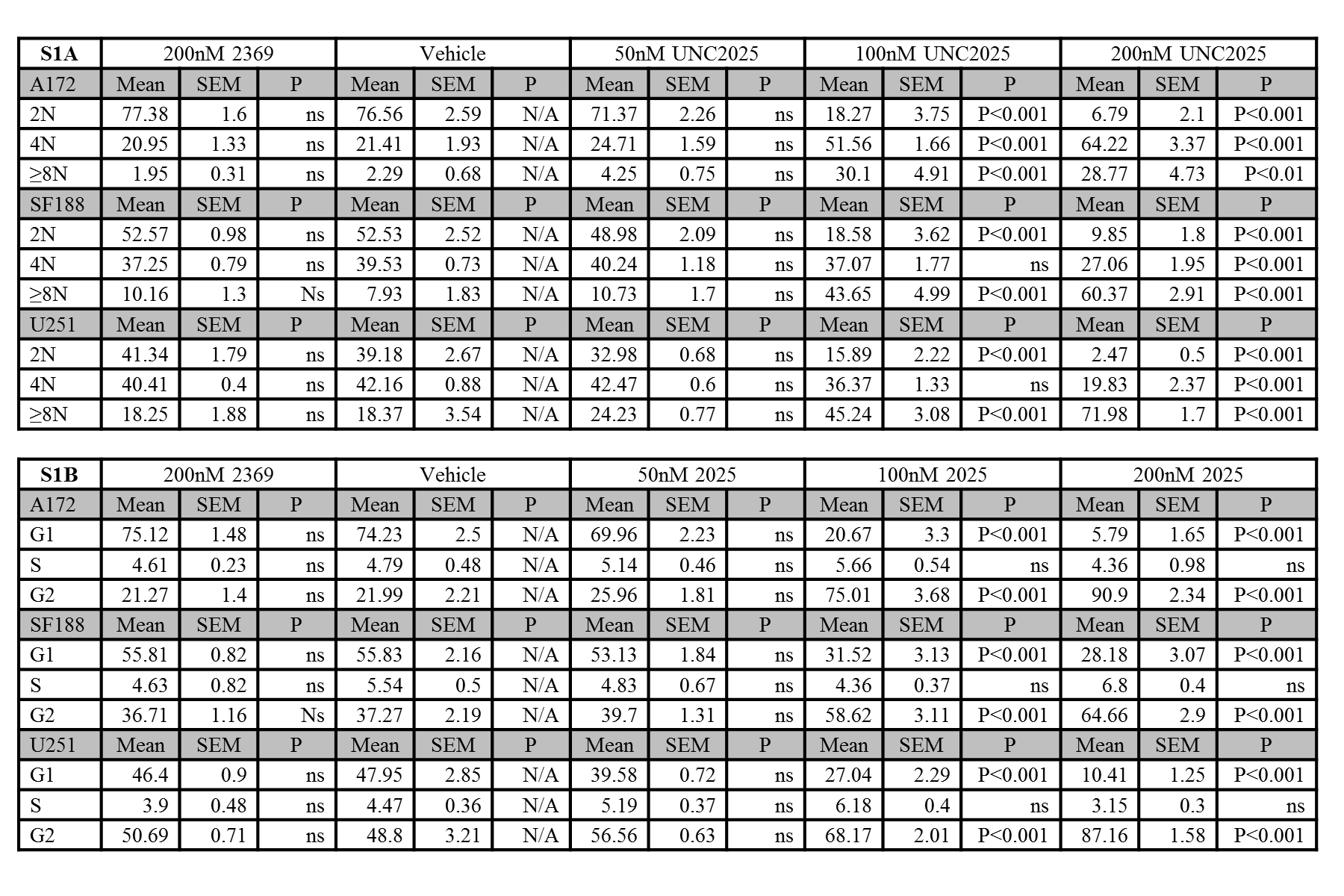

Supplement: S1 Table — A172, SF188, and U251 were cultured with UNC2025, UNC2369 or DMSO vehicle for 48 hours, then stained with Pro-Po-1 and propidium iodide and analyzed by flow cytometry. (A) Percentages of cells with 2N, 4N, and ≥8N DNA content, determined by 7-AAD staining. (B) Percentages of cycling cells in G0/G1, S, and G2/M phases of the cell cycle were determined based on DNA content indicated by 7-AAD staining. Polyploid cells containing >4N DNA content were excluded from the analysis. Mean values and standard errors were derived from 3 independent experiments. Statistical differences relative to vehicle-treated cultures were determined using 1-sided ANOVA. (TIF) [file pone.0165107.s001.tif]

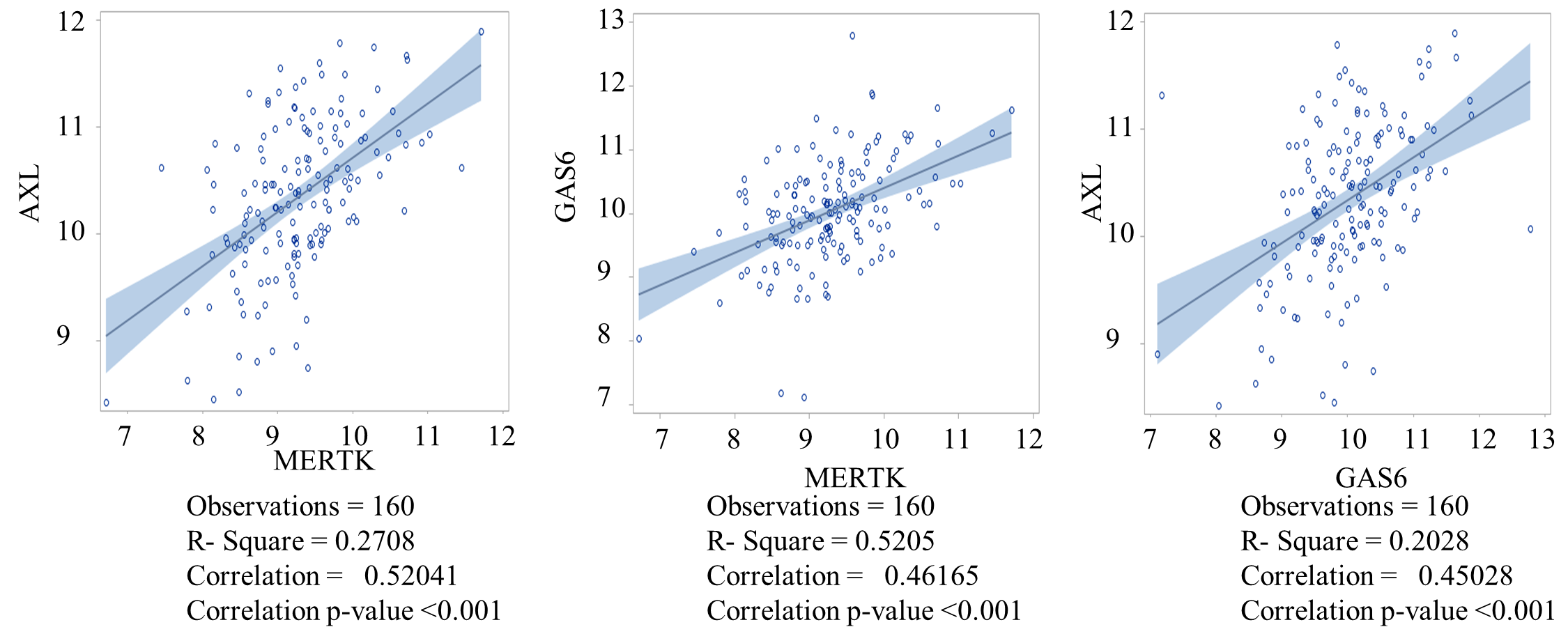

Supplement: S1 Fig — Scatter plots showing significant correlations between expression of MERTK and AXL (left panel), MERTK and GAS6 (middle panel), and AXL and GAS6 (right panel) in GBM patient samples from the TCGA database. (TIF) [file pone.0165107.s002.tif]

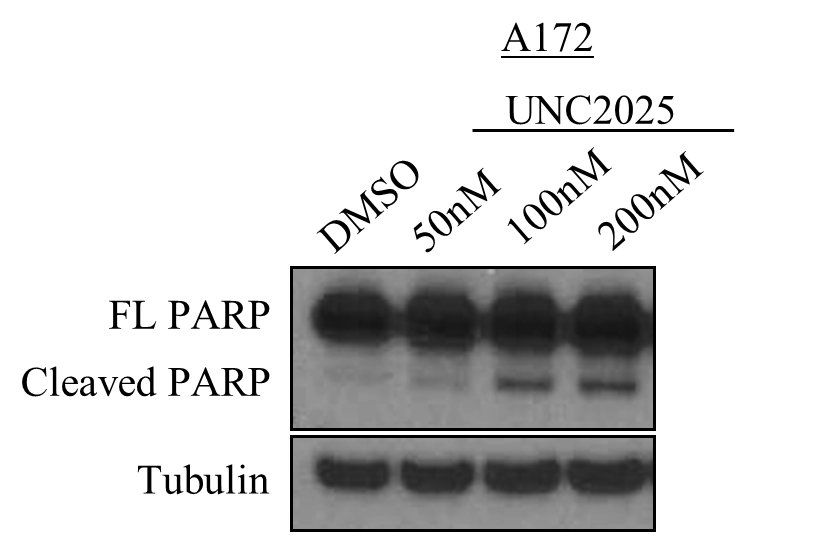

Supplement: S2 Fig — A172 cells were cultured with UNC2025 (50nM, 100nM, and 200nM) for 24 (top panels) or 48 (bottom panels) hours. Whole cell lysates were prepared and the indicated proteins were detected by immunoblot. Images are representative of two independent experiments. (FL = Full length). (TIF) [file pone.0165107.s003.tif]

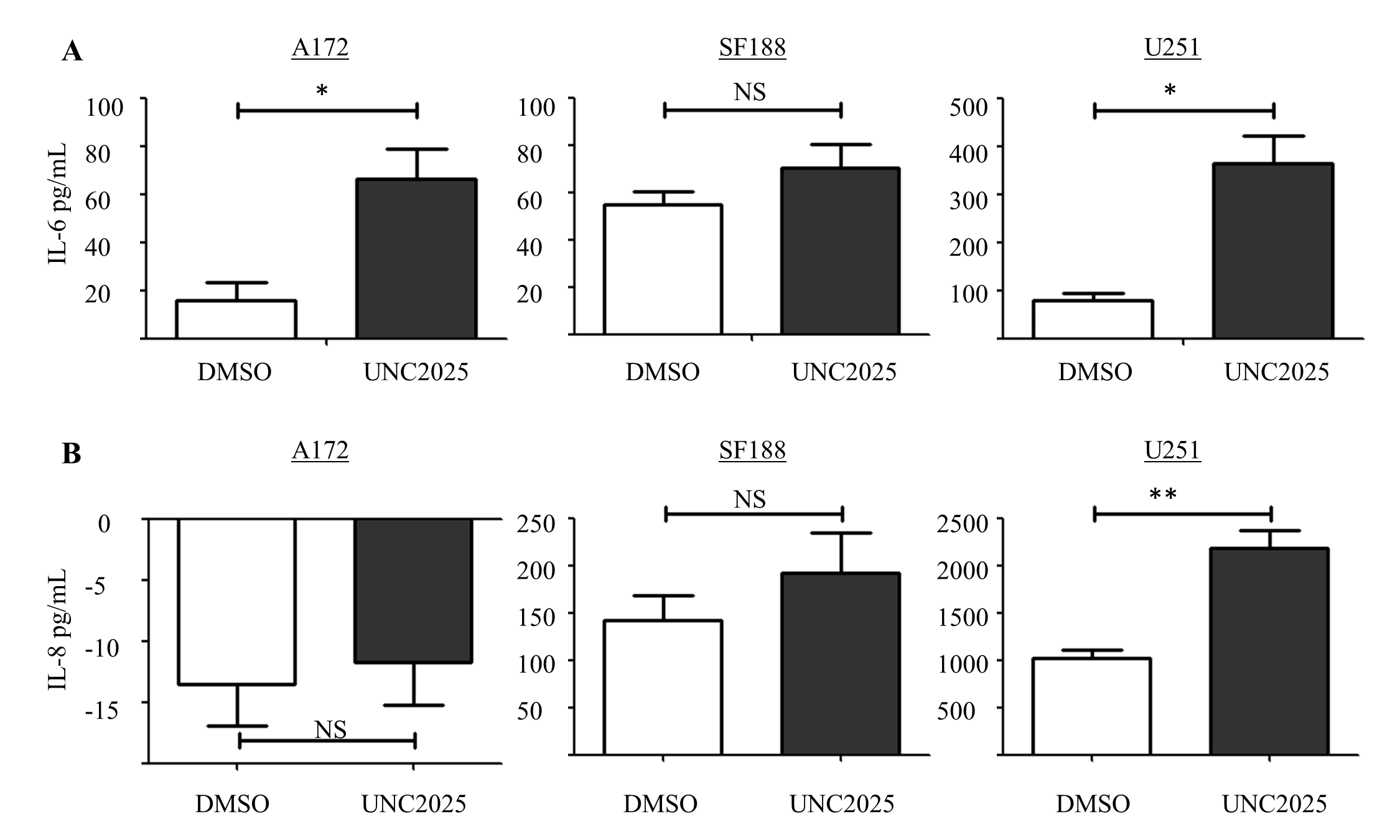

Supplement: S3 Fig — The A172, SF188, and U251 cell lines were cultured with 200nM UNC2025 for 5 days, then media was collected and IL-6 and IL-8 proteins were quantitated by ELISA. Mean values and standard errors derived from 3 independent experiments are shown. (*p<0.05, **p<0.01, 1-sided ANOVA) (TIF) [file pone.0165107.s004.tif]

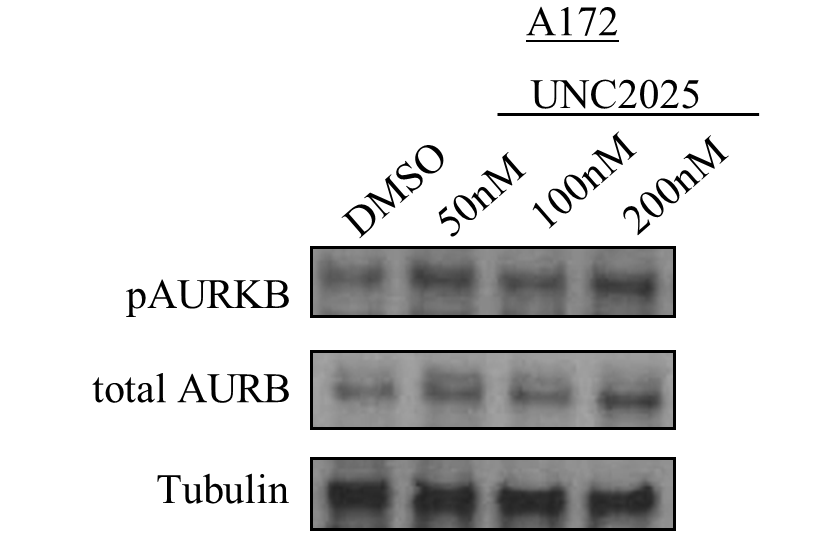

Supplement: S4 Fig — A172 cells were treated with UNC2025 or vehicle for one hour and lysates were prepared. Phosphorylated (denoted by p) and total Aurora Kinase B were detected by immunoblot. Tubulin is shown as a loading control. Images are representative of two independent experiments. (TIF) [file pone.0165107.s005.tif]

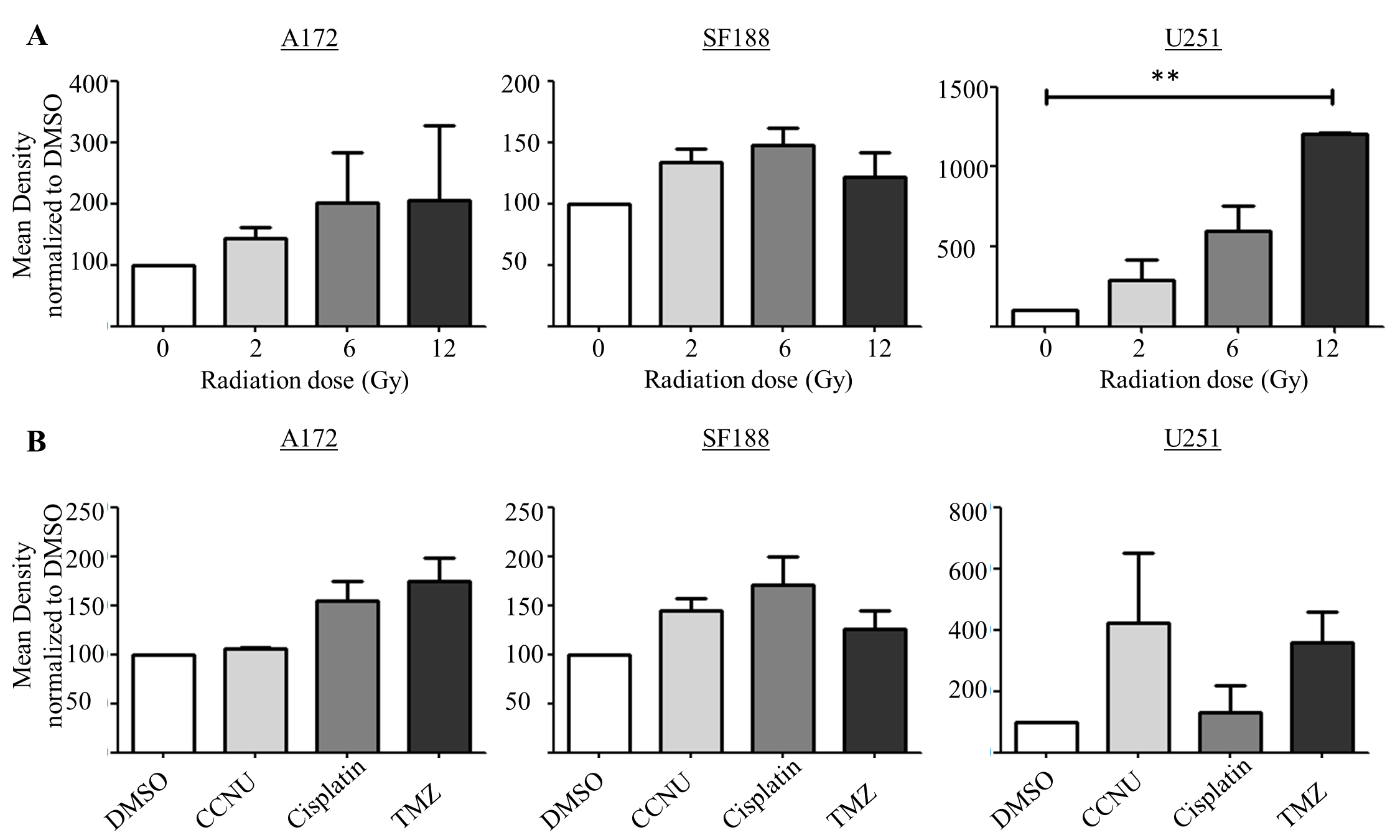

Supplement: S5 Fig — Densitometry was used to quantitate immunoblots derived from cells treated with radiation (A) or cytotoxic chemotherapy (B) as depicted in Fig 6. Mean values and standard errors derived from 2–4 independent experiments are shown. (**p<0.01, 1-sided ANOVA) (TIF) [file pone.0165107.s006.tif]

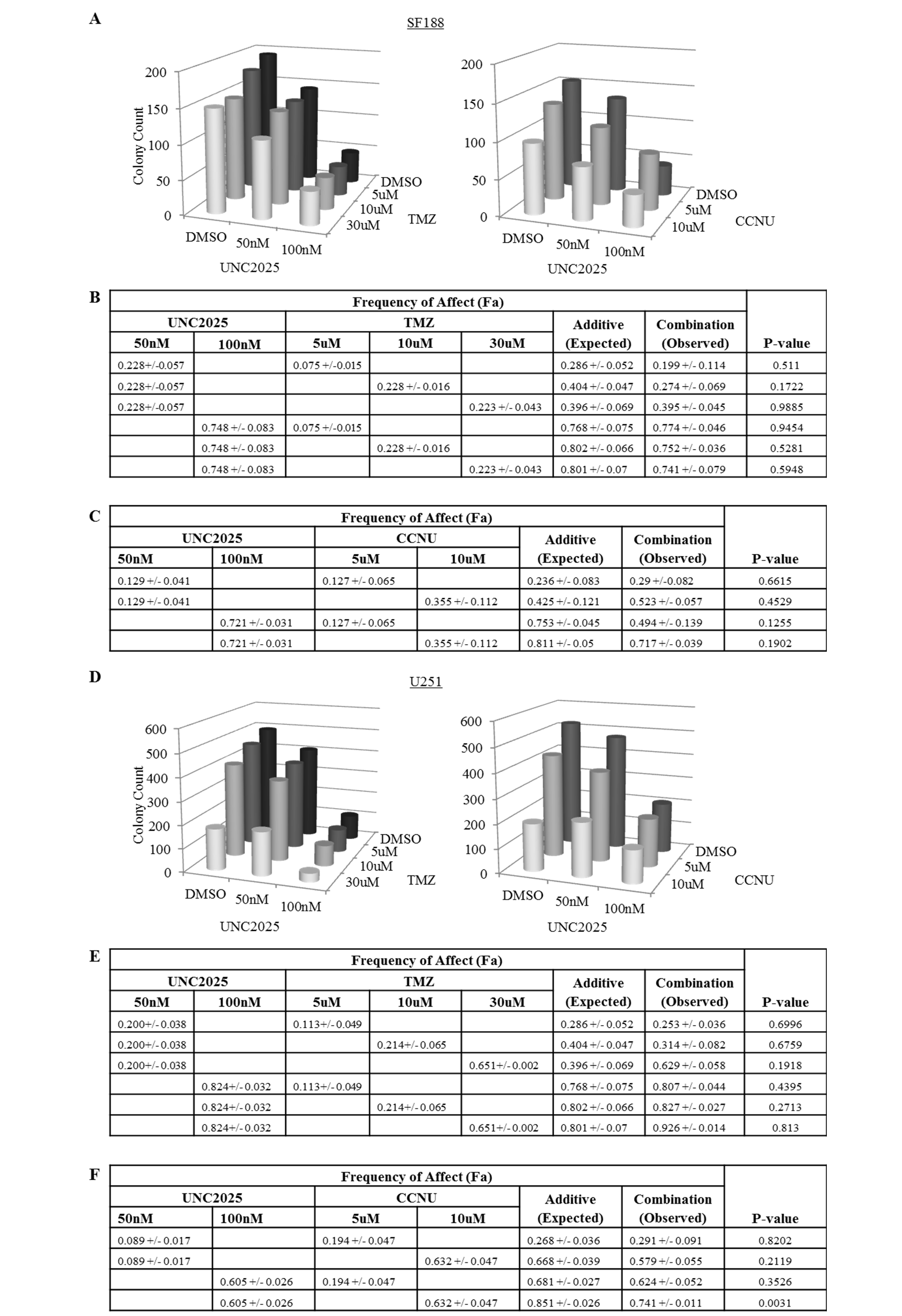

Supplement: S6 Fig — SF188 (A-C) and U251 (D-F) were cultured with UNC2025 and/or temozolomide or lomustine (CCNU) for 9 days. Colonies were fixed and stained with crystal violet in methanol, then counted. The expected frequency of affect (Fa) for an additive interaction was determined using the Bliss additivity model [32] and is shown (Additive). Statistically significant (p value < 0.05, student’s paired t test) increases in the observed Fa mediated by UNC2025 plus chemotherapy (Combination) relative to the values expected for an additive interaction were not observed, indicating additive interactions. Mean values and standard errors were derived from 4–6 independent experiments. (TIF) [file pone.0165107.s007.tif]
